# Widespread use of incorrect PCR ramp rate negatively impacts multidrug-resistant tuberculosis diagnosis (MTBDR*plus*)

**DOI:** 10.1038/s41598-018-21458-y

**Published:** 2018-02-16

**Authors:** B. Derendinger, M. de Vos, R. R. Nathavitharana, T. Dolby, J. A. Simpson, P. D. van Helden, R. M. Warren, G. Theron

**Affiliations:** 1 DST/NRF Centre of Excellence for Biomedical Tuberculosis Research, SA MRC Centre for Tuberculosis Research, Division of Molecular Biology and Human Genetics, Faculty of Medicine and Health Sciences, Stellenbosch University, Cape Town, South Africa; 2 Division of Infectious Diseases, Beth Israel Deaconess Medical Center, Harvard Medical School, Boston, MA 02215 USA; 3 National Health Laboratory Services, Cape Town, South Africa

## Abstract

The scale-up of rapid drug resistance testing for TB is a global priority. MTBDR*plus* is a WHO-endorsed multidrug-resistant (MDR)-TB PCR assay with suboptimal sensitivities and high indeterminate rates on smear-negative specimens. We hypothesised that widespread use of incorrect thermocycler ramp rate (speed of temperature change between cycles) impacts performance. A global sample of 72 laboratories was surveyed. We tested 107 sputa from Xpert MTB/RIF-positive patients and, separately, dilution series of bacilli, both at the manufacturer-recommended ramp rate (2.2 °C/s) and the most frequently reported incorrect ramp rate (4.0 °C/s). *Mycobacterium tuberculosis-*complex DNA (TUB-band)-detection, indeterminate results, accuracy, and inter-reader variability (dilution series only) were compared. 32 respondents did a median (IQR) of 41 (20–150) assays monthly. 78% used an incorrect ramp rate. On smear-negative sputa, 2.2 °C/s vs. 4.0 °C/s improved TUB-band positivity (42/55 vs. 32/55; p = 0.042) and indeterminate rates (1/42 vs. 5/32; p = 0.039). The actionable results (not TUB-negative or indeterminate; 41/55 vs. 28/55) hence improved by 21% (95% CI: 9–35%). Widespread use of incorrect ramp rate contributes to suboptimal MTBDR*plus* performance on smear-negative specimens and hence limits clinical utility. The number of diagnoses (and thus the number of smear-negative patients in whom DST is possible) will improve substantially after ramp rate correction.

## Introduction

There were ~10.4 million reported cases of tuberculosis (TB) and 1.7 million deaths from TB in 2016. Only 22% of the ~490 000 new cases of multidrug-resistant (MDR-) TB in 2016 were diagnosed^1^. Drug-susceptibility testing (DST) has relied on culture for phenotypic and molecular testing (indirect testing)^2^. Earlier drug resistance diagnosis through rapid sputum testing (direct testing) can facilitate early effective treatment initiation^3^ and help render patients non-infectious^4^. This can disrupt transmission^5^; a key driver of MDR-TB^6^ that results in poor patient outcomes and substantial costs^1,7^.

GenoType MTBDR*plus*^8^ (Hain Lifescience, Germany) is a rapid PCR line probe assay for *Mycobacterium tuberculosis-*complex DNA (reported as TUB-band-positive) and rifampicin- and isoniazid-resistance. MTBDR*plus* is World Health Organization- (WHO)^9^ and Centers for Disease Control and Prevention-endorsed^10^. Many countries have incorporated MTBDR*plus* into national diagnostic algorithms^11^. MTBDR*plus* involves the amplification of regions within the *M*. *tuberculosis* genome and their colorimetric visualisation by hybridisation to membrane-bound probes. Despite the manufacturer’s recommendation for use in smear-negative specimens, evidence to support MTBDR*plus* in this context is relatively weak and heterogeneous, with studies describing sensitivities ranging from 40–100% and indeterminate rates ranging from 0.5–14.5%^12–18^. This limited data to support use in smear-negative specimens restricts MTBDR*plus*’s utility in high burden, HIV-endemic settings^19^. The WHO endorsement for direct MTBDR*plus* testing is hence for smear-positive specimens only^20^.

The latest iteration of MTBDR*plus* (version 2) was designed to have improved sensitivity on specimens, irrespective of smear status, and culture isolates. MTBDR*plus*’s follow-on test for second-line resistance (MTBDR*sl*; Hain Lifescience, Germany) is based on similar principles and also WHO-endorsed^9,16,21,22^.

MTBDR*plus* requires thermocycling to amplify DNA. The manufacturer recommends a ramp rate (speed of temperature change between cycles) of ≤ 2.2 °C/s^8^, which the thermocycler they sell (the GTC-cycler) is capable of. Laboratories can use their own thermocyclers, however, these thermocyclers may have different default ramp rates or, in cheaper models, may not permit ramp rate to be changed. None of the studies in a recent systematic review and meta-analysis of MTBDR*plus* report ramp rate and few studies reported rates of TUB-band positivity^16,23^. If an assay is TUB-band-negative, susceptibility results cannot, per the manufacturer’s recommendation, be reported^8^ and studies that do not report TUB-band positivity rates do not provide a complete characterisation of test performance.

We hypothesised that suboptimal sensitivities and high indeterminate rates reported for MTBDR*plus* on smear-negative specimens^12–17^ were partly associated with incorrect ramp rate. If this phenomenon is widespread, it may explain a major limitation in the routine diagnosis of MDR-TB, for which MTBDR*plus* is the only commercially available molecular assay. This could result in large numbers of possible MDR-TB diagnoses being missed, exacerbate diagnostic delay, and will have implications for diagnostic algorithms (e.g., confirmation of Xpert-indicated rifampicin-resistance, detection of rifampicin or isoniazid mono-resistance), clinical practice (e.g., detection of acquired resistance during treatment monitoring), and research studies (e.g., MDR-TB drug trials that need to rapidly screen patients).

## Methods

### Ethics statement

This study was approved by the Health Research Ethics Committee of Stellenbosch University (N09–11–296) and done in accordance with these relevant guidelines and regulations. Permission was granted by the institutional review board (IRB) to access anonymised residual specimens collected as part of routine diagnostic practice and thus patient informed consent was waived.

### Survey of diagnostic and research laboratories

An invitation to an online survey was sent to 74 laboratories using MTBDR*plus* identified from a recent systematic review and meta-analysis^16^, expert consultation, the Global Laboratory Initiative, the Global Health Delivery network, and FIND. We placed no restrictions on the type of facility or country that could respond. Initial non-responders were emailed at least a further three times. Questions included country, average number of MTBDR*plus* assays per month, primary purpose of the assay, specimen smear status, models of thermocyclers, whether the thermocycler permitted ramp rate to be changed, and the MTBDR*plus* ramp rate used (the full questions are listed in the supplement). Permission was obtained from respondents to use their anonymised data for publication.

### Specimen collection and decontamination

107 de-identified sputa consecutively submitted to an accredited government quality-assured (South African National Accreditation System) laboratory in Cape Town, South Africa were collected. Sputa were from patients with symptoms of TB who were, using a separate paired specimen, Xpert MTB/RIF (Xpert)-positive for TB and rifampicin-susceptible or -resistant. Sputa were decontaminated with NaOH-N-Acetyl-L-Cysteine (1% final concentration)^24^. Each decontaminated sediment had ~50 µl used for Auramine-O^25^ smear microscopy and, if the paired specimen was Xpert rifampicin-resistant, ~500 µl used for culture for DST. 52 sputa were smear-positive (26 Xpert-rifampicin resistant, 26 Xpert-rifampicin susceptible) and 55 smear-negative (39 Xpert-rifampicin resistant, 16 Xpert-rifampicin susceptible). The sediments were stored at 4 °C for 2–3 days prior to transport to Stellenbosch University for DNA extraction.

### Impact of thermocycler ramp rate on MTBDR*plus* performance in clinical specimens

DNA was extracted from sediments using the GenoLyse kit (Hain Lifescience, Germany)^8^. DNA was amplified using two ramp rates: the manufacturer-recommended ramp rate (2.2 °C/s), and 4.0 °C/s, the most frequently used incorrect ramp rate in the survey, using a CFX96 (Bio-Rad, United States), which was the only machine available with a customisable ramp rate. This instrument undergoes annual servicing and calibration by the manufacturer. Hybridisation was done with the GT-Blot 48 (Hain Lifescience, Germany)^26^. An experienced reader interpreted bands in a blinded manner.

### Impact of thermocycler ramp rate on MTBDR*plus* performance in a dilution series

A drug-susceptible strain (H37Rv, ATCC 25618) and a phenotypically-confirmed clinical MDR strain (with known *rpoB*, *katG*, and *inhA* promoter SNPs) were grown to mid-exponential phase in Middlebrook 7H9 media (Becton Dickinson, United States) supplemented with Middlebrook Oleic Albumin Dextrose Catalase supplement (Becton Dickinson, United States). Colony counts after incubation on Middlebrook 7H10 media (Becton Dickinson, United States) for 21 days at 37 °C were done. This experiment was done in triplicate. MTBDR*plus* was done on dilutions of 10^2^, 10^3^ and 10^4^CFU/ml in phosphate buffer with 0.025% Tween 80. 10^4^CFU/ml corresponds approximately to smear-positivity^27^ and the lower concentrations in the dilution series correspond to paucibacillary smear-negative disease (i.e., the patients we hypothesise ramp rate to impact the most). The CFX96 machine with ramp rates of 2.2 °C/s or 4.0 °C/s was used. An experienced reader interpreted bands in a blinded manner.

### Assessment of inter-reader variability

MTBDR*plus* strips from the dilution series were interpreted by two experienced technicians in a blinded manner. Variability between readers (individual banding patterns, final diagnostic classifications) was assessed. When a strip is interpreted, a banding call determination is made if a specific band is present or absent; whereas a diagnostic call (susceptibility or resistance to rifampicin and/or isoniazid) is based on the overall banding pattern. Hence, banding patterns may change but not the diagnostic call. Excluding the conjugate and amplification control bands, gene loci control bands, and including the TUB-band, gene-specific wildtype and mutant bands, there are 22 possible bands per strip that we included in our analysis for the comparison of banding pattern readability.

### Classification of MTBDR*plus* results

A positive result for *M*. *tuberculosis-*complex DNA was defined as the presence of the TUB-band with the amplification and conjugation control bands. Sensitivity for *M. tuberculosis*-complex DNA was calculated using a paired MGIT960 liquid culture (Becton Dickinson, United States) result from the national laboratory as a reference standard. A strip was classified as indeterminate if the amplification or conjugate control bands were absent but any other bands were present. A drug indeterminate result was defined as the absence of any locus control band (*rpoB*, *katG* and *inhA*) on a TUB-band-positive strip. A result was classified as actionable if the strip was TUB-band-positive and not indeterminate for any drugs.

### Statistical analyses

The two sample test of proportion was used for comparisons between proportions, and McNemar’s test was used to calculate differences in sensitivity or indeterminate rates across ramp rates for paired data. We used the percent improvement in actionable results (calculated from our clinical specimen experiment, 21%) to estimate the number of additional TUB-band-positive diagnoses (and MDR-TB diagnoses) in survey respondents who said they tested smear-negative specimens. For this calculation, we assumed 1) the volume of assays done by the respondent was evenly spread across input material types (e.g., a respondent doing 100 MTBDR*plus* assays per month does ~33 smear-positive, smear-negative, and isolates; we unfortunately did not retrieve specific data on the monthly volume of smear-negative specimens only), 2) the MDR-TB prevalence in smear-negative specimens corresponded to the overall WHO estimate for the respondent’s country, and 3) ramp rate changes would equally affect resistance and susceptibility detection. We used GraphPad Prism version 6.0 (GraphPad Software) and Stata version 14 (StataCorp) software. All statistical tests are 2-sided at α = 0.05.

### Data availability

The datasets generated during and/or analysed during the current study are available from the corresponding author on reasonable request.

## Results

### Survey

**Figure 1:**
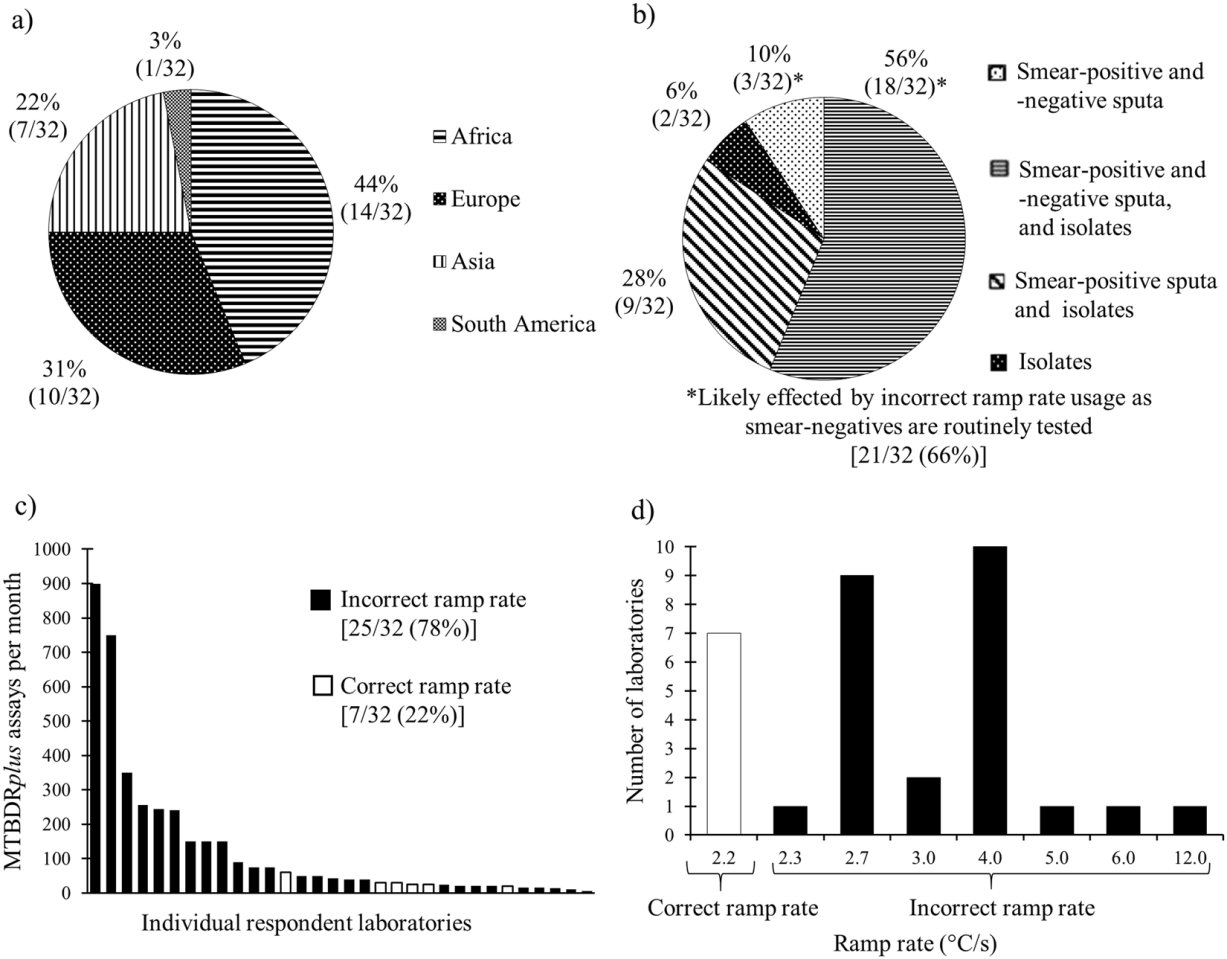
Survey results showing breakdowns of (**a**) the geographical locations of laboratory survey respondents, (**b**) type of input material used (smear status and/or culture isolate) for MTBDR*plus*, (**c**) amount of MTBDR*plus* assays done per laboratory each month and (**d**) ramp rates used by respondents

Laboratory respondents were geographically diverse and often tested smear-positive and smear-negative specimens, as well as culture isolates (Fig. 1a,b). Our survey response rate was 32/72 (44%). Respondents did a median [interquartile range (IQR)] of 41 (20–150) MTBDR*plus* assays per month (Fig. 1c). 18/32 (56%) of respondents used MTBDR*plus* for both routine diagnosis and research. Critically, 25/32 (78%) of respondents used an incorrect ramp rate (Fig. 1d), ranging from 2.3–12.0 °C/s. Of respondents that tested smear-negative specimens (21/32; 66%), 16/21 (76%) used an incorrect ramp rate. Stratified by continent, 3/14 (21%) African, 1/7 (14%) Asian, 3/10 (30%) European and 0/1 (0%) South American laboratories used the correct ramp rate. 19/32 (59%) of respondents indicated that they could set the correct ramp rate by adjusting their thermocycler, whereas the remainder did not use thermocyclers with customisable ramp rates (Table 1). 10/32 (31%) laboratories did MTBDR*plus* only and 22/32 (69%) did MTBDR*plus* and MTBDR*sl*.

**Table 1:** Answers to survey questions stratified by continent.

Country	MTBDR*plus*/month	MTBDR*plus* use	MTBDR*plus* sample type	MTBDR*sl*/month	MTBDR*sl* use	MTBDR*sl* sample type	Other LPAs	Thermocycler manufacturer	Thermocycler	Ramp rate (°C/s)	Customisable ramp rate	Pre-screening Xpert MTB/RIF
**Africa [14 laboratories; median (IQR) of 46 (20–241) MTBDR** ***plus*** **assays per month]**												
South Africa	900	Diagnosis	Smear-positive, smear-negative, isolates	50	Diagnosis	Smear-positive, smear-negative, isolates	N/A	Applied Biosystems	SimpliAmp	4.0	Yes	Yes
Swaziland	350	Diagnosis, research	Smear-positive, isolates	0	Diagnosis, research	Smear-positive, isolates	N/A	Applied Biosystems	2720 Thermal Cycler	2.7	No	Yes
South Africa	245	Diagnosis	Smear-positive, isolates	0	Diagnosis	Smear-positive, isolates	CM/AS	Applied Biosystems	ABI	4.0	Yes	Yes
Kenya	240	Diagnosis, research	Smear-positive, smear-negative	0	Research	Smear-positive	CM/AS	Applied Biosystems	GeneAmp 9700	2.3	Yes	Yes
South Africa	150	Diagnosis, research	Isolates	15	Diagnosis	Isolates	N/A	Applied Biosystems	ABI	6.0	Yes	Yes
Ethiopia	75	Diagnosis, research	Smear-positive, isolates	60	Diagnosis, research	Smear-positive, smear-negative, isolates	CM/AS	Applied Biosystems	2720 Thermal Cycler	2.7	No	Yes
South Africa	50	Diagnosis	Smear-positive, smear-negative, isolates	45	Diagnosis	Smear-positive, smear-negative, isolates	CM	Applied Biosystems	Proflex PCR system	4.0	Yes	No
Nigeria	42	Diagnosis, research	Smear-positive, smear-negative, isolates	27	Diagnosis, research	Smear-positive, smear-negative, isolates	CM/AS	Applied Biosystems	2720 Thermal Cycler	2.7	No	Yes
South Africa	40	Diagnosis, research	Isolates	0	Diagnosis, research	Isolates	CM	Applied Diagnosis	Various	4.0	Yes	Yes
**Nigeria**	**25**	**Diagnosis**	**Smear-positive, smear-negative, isolates**	**35**	**Diagnosis**	**Smear-positive, smear-negative, isolates**	**CM**	**Hain Lifescience**	**GTQ-cycler 96**	**2.2**	**Yes**	**Yes**
**Cameroon**	**20**	**Diagnosis, research**	**Smear-positive, smear-negative**	**20**	**Diagnosis, research**	**Smear-positive, smear negative**	**CM/AS**	**Applied Biosystems**	**GeneAmp 9700**	**2.2**	**Yes**	**Yes**
**Botswana**	**20**	**Diagnosis, research**	**Smear-positive, isolates**	**20**	**Diagnosis, research**	**Smear-positive, isolates**	**CM**	**Hain Lifescience**	**GTQ-cycler 96**	**2.2**	**Yes**	**No**
South Africa	20	Diagnosis, research	Smear-positive, smear-negative, isolates	0	Diagnosis, research	Smear-positive, smear-negative, isolates	CM	Bio-Rad	Various	4.0	No	No
Côte d’Ivoire	14	Diagnosis, research	Smear-positive, isolates	4	Diagnosis, research	Smear-positive, isolates	N/A	Applied Biosystems	2720 Thermal Cycler	2.7	No	Yes
**Asia [7 laboratories; median (IQR) of 150 (15–256) MTBDR** ***plus*** **assays per month]**												
Azerbaijan	750	Diagnosis, research	Smear-positive, isolates	0	Diagnosis, research	Smear-positive, isolates	N/A	Unknown	Unknown	12.0	No	Yes
Kyrgyzstan	256	Diagnosis	Smear-positive, smear-negative, isolates	37	Diagnosis	Smear-positive	N/A	Biometra	Tprofessional	3.0	Yes	Yes
Bangladesh	150	Diagnosis	Smear-positive, smear-negative, isolates	120	Diagnosis	Smear-positive, smear-negative, isolates	N/A	Applied Biosystems	2720 Thermal Cycler	2.7	No	Yes
India	150	Diagnosis	Smear-positive, smear-negative, isolates	0	Diagnosis	Smear-positive, isolates	CM	Bio-Rad	Thermal Cycler T100	4.0	No	No
**Pakistan**	**30**	**Diagnosis, research**	**Smear-positive, smear-negative, isolates**	**2**	**Research**	**Isolates**	**CM/AS**	**Hain Lifescience**	**GTQ-cycler 96**	**2.2**	**Yes**	**Yes**
Myanmar	15	Diagnosis	Smear-positive, smear-negative, isolates	10	Diagnosis	Smear-positive, smear-negative, isolates	N/A	Applied Biosystems	2720 Thermal Cycler	2.7	No	Yes
Thailand	5	Diagnosis	Smear-positive, smear-negative	0	Research	Isolates	N/A	Applied Biosystems	2720 Thermal Cycler	2.7	No	No
**Europe [10 laboratories; median (IQR) of 35 (23–64) MTBDR** ***plus*** **assays per month]**												
Denmark	90	Diagnosis, research	Smear-positive, smear-negative, isolates	5	Diagnosis, research	Smear-positive, isolates	CM/AS	Applied Biosystems	SimpliAmp	4.0	Yes	No
Belarus	75	Diagnosis	Smear-positive, smear-negative, isolates	80	Diagnosis	Smear-positive, smear-negative, isolates	CM/AS	Applied Biosystems	2720 Thermal Cycler	2.7	No	Yes
**Moldova**	**60**	**Diagnosis, research**	**Smear-positive, smear-negative, isolates**	**24**	**Diagnosis, research**	**Smear-positive, smear-negative**	**N/A**	**Hain Lifescience**	**GTQ-cycler 96**	**2.2**	**Yes**	**Yes**
Belgium	50	Diagnosis, research	Smear-positive, isolates	30	Diagnosis, research	Smear-positive, isolates	Nipro	Biometra	Tprofessional	3.0	Yes	Yes
Belarus	40	Diagnosis	Smear-positive, isolates	0	Diagnosis	Smear-positive, isolates	CM	Applied Biosystems	2720 Thermal Cycler	2.7	No	Yes
**Germany**	**30**	**Diagnosis**	**Smear-positive, smear-negative, isolates**	**10**	**Diagnosis**	**Smear-positive, smear-negative, isolates**	**N/A**	**Hain Lifescience**	**GTQ-cycler 96**	**2.2**	**Yes**	**Yes**
Denmark	25	Diagnosis, research	Smear-positive, smear-negative, isolates	4	Diagnosis, research	Smear-positive, smear-negative, isolates	CM/AS	Applied Biosystems	SimpliAmp	4.0	Yes	No
France	25	Diagnosis	Smear-positive, smear-negative, isolates	17	Diagnosis	Smear-positive, smear-negative, isolates	CM/AS, NTM-DR	Bio-Rad	Various	4.0	Yes	No
**Italy**	**15**	**Diagnosis, isolates**	**Smear-positive, isolates**	**15**	**Diagnosis, research**	**Smear-positive, isolate**	**N/A**	**Hain Lifescience**	**GTQ-cycler 96**	**2.2**	**Yes**	**No**
Spain	10	Diagnosis	Smear-positive, smear-negative, isolates	2	Diagnosis	Smear-positive, smear-negative, isolates	Inno-Lipa	Applied Biosystems	Various	4.0	Yes	No
**South America (1 laboratory; 20 MTBDR** ***plus*** **assays per month)**												
Brazil	20	Diagnosis, research	Smear-positive, smear-negative, isolates	0	Diagnosis, research	Smear-positive, smear-negative, isolates	MOTT ID	Bio-Rad	C1000 Touch	5.0	Yes	Yes
**Overall [32 laboratories; median (IQR) of 41 (20–150) MTBDR** ***plus*** **assays per month]**												
	3987			632		16/32 respondents test smear-negative specimens and are hence likely affected by incorrect ramp rate usage				7/32 used the correct ramp rate	20/32 have a customisable ramp rate	22/32 use Xpert as a pre-screen

LPA - Line probe assay; HCV – HCV Genotype 2.0 Assay (LiPA); CM-Genotype Mycobacterium CM Ver 2.0; NTM-DR – GenoType NTM-DR Ver 1.0;

AS - Genotype Mycobacterium AS Ver 1.0; IQR - Interquartile Range; Xpert – Xpert MTB/RIF assay.

Survey questions are in the supplement. Text in bold refers to laboratories using the manufacturer-recommended ramp rate.

### Performance of MTBDR*plus* at different ramp rates on clinical specimens

In smear-positive specimens (n = 52), TUB-band detection was 100% irrespective of ramp rate, whereas in smear-negative specimens TUB-band detection was 76% (42/55) at 2.2 °C/s and 58% (32/55) at 4.0 °C/s (p = 0.042). Smear-positive specimens had no indeterminate results. For smear-negative specimens, of the 42 TUB-band positives at 2.2 °C/s, 1 (2%) was indeterminate for isoniazid whereas at 4.0 °C/s, 5/32 (16%) TUB-positive specimens were indeterminate for isoniazid (p = 0.093) (Table 2). There were no indeterminate results for rifampicin in the clinical specimens, regardless of ramp rate. Thus, for smear-negative specimens, an actionable result could not be generated at 2.2 °C/s for 14/55 (13 TUB-negatives + 1 indeterminate result for isoniazid; 25%) specimens and 28 (23 TUB-negatives + 5 indeterminate results for isoniazid; 51%) specimens at 4.0 °C/s (p = 0.006). Hence, ramp rate correction resulted in a 21% (95% CI: 9–35%; p < 0.0001) increase in the number of diagnoses in smear-negative specimens. If we apply this increase to the volume of testing reported by our 16 respondent laboratories that test smear-negative specimens, we would expect an additional ~89 TUB-band positive tests per month in smear-negative specimens that, at each respondent’s local MDR-TB prevalence, should translate into ~7 additional MDR-TB diagnoses overall amongst the respondents.

**Table 2:** Performance of Genotype MTBDR*plus* at different ramp rates for the detection of *M*. *tuberculosis*-complex DNA (TUB-band), stratified by smear status, when done directly on sputa from Xpert MTB/RIF-positive patients. Genolysed extract from each specimen was tested at each ramp rate. TUB-band detection and the rate of indeterminates worsened in smear-negative specimens with use of the incorrect ramp rate.

Smear microscopy status	Ramp rate (°C/s)	TUB-band positive (%)	Determinate (%)	Indeterminate (%)
Positive n = 52	4.0	52/52 (100)	52/52 (100)	0/52 (0)
	2.2^*^	52/52 (100)	52/52 (100)	0/52 (0)
Negative n = 55	4.0	32/55 (58)	27/32 (84)	5/32 (16)
	2.2^*^	42/55 (76) (p = 0.042)	41/42 (98) (p = 0.164)	1/42 (2) (p = 0.039)

P-values are for comparisons between ramp rates for smear-negative specimens.

^*^Manufacturer-recommended ramp rate.

### Performance of MTBDR*plus* at different ramp rates on dilution series of bacilli

Each of the three technical replicates for each strain in the dilution series (10^2^, 10^3^ and 10^4^CFU/ml) were TUB-band-positive and there were no indeterminate results, irrespective of ramp rate. At 4.0 °C/s, the drug-susceptible strain gave a false-positive rifampicin-resistance result in a 10^2^CFU/ml replicate, however, at higher concentrations all results were true-susceptible. Overall, bands at 2.2 °C/s were subjectively interpreted as being darker, clearer, and more distinct than those at 4.0 °C/s by the experienced readers.

### Assessment of inter-reader agreement on dilution series

Banding and diagnostic calls differed between readers and were most pronounced at 10^2^CFU/ml (Table 3). Of the 198 possible non-control bands in the dilution series experiment for the drug-susceptible strain, readers disagreed on 1% (2/198) of bands at 4.0 °C/s but none at 2.2 °C/s (p = 0.156). For the MDR strain, there were 6/198 (3%) band differences between readers at 4.0 °C/s and 1/198 (0.5%) differences at 2.2 °C/s (p = 0.057). At 4.0 °C/s, one reader reported one replicate of the MDR strain as false-susceptible to rifampicin at 10^2^CFU/ml and the same strain as TUB-band-negative at 10^3^CFU/ml (the other reader read both these strips correctly).

**Table 3:** Comparison of banding calls and diagnostic calls from two experienced readers of MTBDRplus done on serial dilutions of *M. tuberculosis* .

	**Ramp rate (°C/s)**	
	**2**.**2** (%)	**4**.**0** (%)
**Banding calls**		
Different band calls between readers for susceptible strain	0/198^*^ (0)	2/198 (1) (p = 0.156)
Different band calls between readers for resistant strain	1/198 (0.5)	6/198 (3) (p = 0.057)
**Diagnostic calls**		
Susceptible strain correctly classified by both readers	18/18 (100)	16/18 (89) (p = 0.146)
Resistant strain correctly classified by both readers	18/18 (100)	16/18 (89) (p = 0.146)

^*^22 bands per strip × three dilutions × three replicates = a total of 198 bands.

P-values are for within-row comparisons between different ramp rates.

## Discussion

Our key findings are: 1) the vast majority of survey respondents, who are globally diverse and do a large volume of MTBDR*plus* assays, use an incorrect ramp rate and this, 2) decreases sensitivity for TB (and hence precludes resistance detection), 3) increases indeterminate rates in smear-negative specimens, and 4) likely increases false-resistance calls and banding pattern disagreement between readers. These findings are of clinical relevance as most respondents used this assay routinely, indicating that incorrect ramp rate usage is likely affecting patient diagnoses.

To the best of our knowledge, ours is the first evaluation of ramp rate on commercial assay performance in the clinical diagnostics literature. Ramp rate has been previously-documented to be important: techniques such as “slowdown PCR”, which are optimised to amplify GC-rich regions with complex secondary structures, use different rates for heating and cooling to improve primer annealing and amplification. Here, ramp rate is critical for the performance of this technique^28^. As *M*. *tuberculosis* is GC-rich and *rpoB* can form secondary structures^29^, it is possible that slower ramp rates help reduce secondary structure formation (e.g., during the transition from denaturation to annealing phases) and thereby result in better detection.

Our survey found the majority of laboratories to use an incorrect ramp rate, despite a lower ramp rate being recommended. About half of respondents could change the ramp rate. Together, this illustrates that incorrect ramp rate usage is likely widespread but, importantly, easily fixable without the purchase of new thermocyclers, which may be prohibitively expensive in high burden settings.

TUB-band detection on smear-negative sputa failed more frequently at incorrect ramp rates. As this band is required before a susceptibility result is reported, drug resistant diagnoses are more likely to be missed at the incorrect ramp rate. Differences in ramp rate may hence partly explain previously reported variation in performance in smear-negative specimens^12–15,17^ however, we only received responses to our queries regarding ramp rate from two studies in the systematic review who used smear-negative specimens.

Although Xpert is often the initial first-line test for rifampicin-resistance, MTBDR*plus* is used for MDR-TB in several high TB-burden countries and to confirm isoniazid-susceptibility. Isoniazid can be included in the new WHO-endorsed MDR-TB second-line regimen^30^. In response to the WHO’s endorsement of the regimen, laboratories are scaling-up MTBDR*sl* capacity for second-line drug resistance testing. MTBDR*sl* is thus of increasing importance, however, we did not include MTBDR*sl* for reasons of cost and feasibility. MTBDR*sl* is nevertheless similar to MTBDR*plus*, has the same recommended ramp rate, and is hence likely similarly adversely impacted. We will validate this in future.

We did not assess the impact of several ramp rates or thermocyclers for reasons of cost and limited clinical specimens, but chose to use the most frequently reported incorrect ramp rate and a machine commonly used in our setting (the survey results showed a large diversity in thermocycler models used, with no predominant model). We did not spike sputa with bacilli as clinical specimens from patients, which we also included, are more suitable (bacilli from patients in sputum are suspended in a mucous matrix rather than bubbles as they are in spiked sputa). Furthermore, spiking was not done at very low concentrations ( < 10^2^ CFU/ml), where the incorrect ramp rate might have more of an impact, however, such concentrations of bacilli are clinically rare and often Xpert-negative (and hence unlikely be tested by MTBDR*plus*). Examination of the impact of ramp rate at lower concentrations might be required for tests that succeed MTBDR*plus* and have higher sensitivity. Finally, despite repeated attempts to survey a wide range of laboratories, it is possible that non-respondents may have different ramp rate usage patterns (e.g., due to less TB or research expertise), which may limit generalisability. This implies our estimated extent of incorrect ramp rate usage is an underestimate.

Our study is the first to investigate ramp rate as a cause of suboptimal MTBDR*plus* performance. We recommend 1) laboratories switch to the manufacturer-recommended ramp rate, 2) the manufacturer makes the recommended ramp rate more prominent in the documentation accompanying the assay, and 3) studies on the line probe assays publish the ramp rate used. Furthermore, we suggest that diagnostic laboratories who have conducted pilot evaluations of MTBDR*plus* on smear-negative specimens and found MTBDR*plus* to have unsatisfactorily high rates of non-actionable results repeat the evaluation if an incorrect ramp rate was originally used.

In conclusion, incorrect ramp rate usage is a widespread problem that negatively affects the diagnostic accuracy of potentially thousands of MTBDR*plus* assays each month. New molecular tests for drug-resistance are critical, however, if they are not done using the correct manufacturer-recommended conditions, performance is compromised and recent promising technical advances (e.g., ability to test smear-negative specimens) will not be fully capitalised upon. Laboratories doing MTBDR*plus* should hence ensure they use the correct thermocycler ramp rate of ≤ 2.2 °C/s.

## Electronic supplementary material

Supplementary Information
