# Supplementary material for: Widespread use of incorrect PCR ramp rate negatively impacts multidrug-resistant tuberculosis diagnosis (MTBDRplus)
Source: Sci Rep. 2018 Feb 16;8:3206. doi: 10.1038/s41598-018-21458-y (PMC5816666; doi:10.1038/s41598-018-21458-y)
Supplement: Supplementary file 1 — Supplementary Information [file 41598_2018_21458_MOESM1_ESM.pdf]

Widespread use of incorrect PCR ramp rate negatively impacts multidrug-resistant tuberculosis diagnosis (MTBDR*plus*)

B Derendinger[1 *], M de Vos[1 *], RR Nathavitharana[2], T Dolby[3], JA Simpson[3], PD van Helden[1], RM Warren[1], G Theron[1†]

Affiliations: [1]DST/NRF Centre of Excellence for Biomedical Tuberculosis Research, SA MRC Centre for Tuberculosis Research, Division of Molecular Biology and Human Genetics, Faculty of Medicine and Health Sciences, Cape Town, Stellenbosch University, South Africa

[2]Division of Infectious Diseases, Beth Israel Deaconess Medical Center, Harvard Medical School, Boston, MA 02215, USA

[3]National Health Laboratory Services, Cape Town, South Africa.

[*]Equal contribution

Hain Lifescience Line Probe Assay (MTBDR*plus* v 2) Survey

1. Name:
2. Name of organisation:
3. Email address:
4. What country are you based in?
5. Over the last 3 months, how many MTBDR*plus* (v2.0) assays would you estimate your laboratory has routinely done per month?
6. Are MTBDR*plus* (v2.0) assays done in your laboratory for:

   Mark only one oval.

   - Routine diagnoses
   - Research use
   - Both
7. Over the last 3 months, how many MTBDR*sl* (v2.0) assays would you estimate your laboratory has routinely done per month?
8. Are MTBDR*sl* (v2.0) assays done in your laboratory for: *

   Mark only one oval.

   - Routine diagnoses
   - Research use
   - Both
9. Does your laboratory perform any other Line Probe Assays? If so, please list.
10. What model(s) of PCR machines does your laboratory use for the MTBDR*plus* (v2.0) and MTBDR*sl* (v2.0) assays?
11. What ramp rate do these PCR machine(s) use?
12. Is the ramp rate customisable on these PCR machines?

    Mark only one oval.

    - Yes
    - No
13. Does your laboratory perform the MTBDR*plus* (v2.0) assay on (more than one option may be selected):

    Check all that apply:

    - Smear-positive samples
    - Smear-negative samples
    - Isolates

14. Does your laboratory perform the MTBDR*sl* (v2.0) assay on (more than one option may be selected):

    Check all that apply.

    - Smear-positive samples
    - Smear-negative samples
    - Isolates

15. Does your laboratory use GeneXpert as a pre-screening tool?

    Mark only one oval.

    - Yes
    - No

16. May we possibly include the aggregated results of this survey in a publication? Names of people and organisations will not be used.

    Mark only one oval.

    - Yes
    - No
